# Altered Jagged1-Notch1 Signaling in Enhanced Dysfunctional Neovascularization and Delayed Angiogenesis After Ischemic Stroke in HFD/STZ Induced Type 2 Diabetes Rats

**DOI:** 10.3389/fphys.2021.687947

**Published:** 2021-07-08

**Authors:** Zhihui Guo, Jia Jia, Yanling Tu, Chang Jin, Cen Guo, Feifei Song, Xuqing Wu, Haifeng Bao, Wei Fan

**Affiliations:** ^1^Department of Neurology, Zhongshan Hospital, Fudan University, Shanghai, China; ^2^Department of Neurology, Shanghai Xuhui District Central Hospital, Shanghai, China; ^3^Department of Neurology, Zhongshan Hospital, Xiamen University, Xiamen, China; ^4^Department of Neurology, Yueyang Hospital of Integrated Traditional Chinese and Western Medicine, Shanghai University of Traditional Chinese Medicine, Shanghai, China

**Keywords:** Jagged1, Notch1, neovascularization, angiogenesis, cerebral ischemia, T2DM

## Abstract

Diabetes exacerbates brain damage in cerebral ischemic stroke. Our previous study has demonstrated that after cerebral ischemia, type 2 diabetes rats displayed worse neurological outcomes, larger cerebral infarction and severer blood-brain barrier disruption. However, our knowledge of the mechanisms of how diabetes impacts the cerebrovascular repair process is limited. This study was aimed to characterize structural alterations and potential mechanisms in brain microvessels before and after ischemic stroke in type 2 diabetic rats treated with high-fat diet and streptozotocin (HFD/STZ). Furtherly, we tested our hypothesis that dysregulated intercellular Jagged1-Notch1 signaling was involved in the dysfunctional cerebral neovascularization both before and after ischemic stroke in HFD/STZ rats. In our study, we found increased yet dysfunctional neovascularization with activated Jagged1-Notch1 signaling in the cerebrovasculature before cerebral ischemia in HFD/STZ rats compared with non-diabetic rats. Furthermore, we observed delayed angiogenesis as well as suppressed Jagged1-Notch1 signaling after ischemic stroke. Our results elucidate the potential mechanisms underlying diabetes-related cerebral microvasculature dysfunction after ischemic stroke.

## Introduction

As of 2019, the global estimate of diabetes prevalence is 9.3% (463 million people), with type 2 diabetes mellitus (T2DM) accounting for approximately 90% of the total (Saeedi et al., 2019). Substantial burden is imposed on both the individual and society as it is associated with multiple complications healthcare management including ischemic stroke (Cannon et al., 2018). Diabetes poses a about four times increase on risk of ischemic stroke (Tun et al., 2017) and ultimately leads to worse long-term functional recovery after stroke (Megherbi et al., 2003) and independently doubles the risk of recurrent stroke (Palacio et al., 2014; Pan et al., 2016).

Due to atherosclerotic lesions of intracranial and extracranial arteries in T2DM, ischemic stroke is generally regarded as a macrovascular complication of diabetes. However, recent evidence has also revealed severe cerebral microvasculature impairment in diabetic patients (Guo et al., 2021). According to previous studies focusing on T2DM patients, diffuse brain atrophy, white matter lesions, microbleeds, and asymptomatic lacunar infarcts have been shown on brain magnetic resonance imaging (MRI) or in postmortem studies (Sima, 2010). These lesions may be clinically asymptomatic if they are single, but more and more single lesion and combinations of lesion types are associated with cognitive and mood disorder, and more impressively, higher risk and poorer prognosis of stroke (Wardlaw et al., 2019). Moreover, altered cerebral microcirculation such as increased blood-brain barrier permeability (Yu et al., 2016), diminished baseline regional cerebral blood flow (CBF), and impaired vasoreactivity have been proposed in diabetes patients (Last et al., 2007; Cui et al., 2017). Collectively, emerging evidence indicates that cerebral microvascular dysfunction in T2DM is one of the key underlying mechanisms of stroke, dementia, and depression (van Sloten et al., 2020).

The clinical evidence mentioned above highlights the demand for basic research focusing on the effect of diabetes on cerebral microvasculature in ischemic stroke. It has been shown that Goto-Kakizaki (GK) rats (Li et al., 2010; Prakash et al., 2012) and db/db mouse (Prakash et al., 2013a) developed enhanced, yet immature, neovascularization in the brain, which may cause the diabetic vessels more vulnerable to reperfusion injury, resulting in greater hemorrhagic transformation (Ergul et al., 2007; Prakash et al., 2012). Furthermore, according to recent reports, cerebrovasculature in peri-infarct regions after an ischemic event was significantly declined in diabetes animals, while controls had compensatory neovascularization (Prakash et al., 2013b). In addition, type 1 diabetes mice exhibited delayed angiogenesis after ischemic stroke (Poittevin et al., 2015), which may also explain why diabetes aggravates ischemic brain injury.

The underlying mechanisms by which diabetes damages the cerebral microvascular network are unclear and may be multi-factorial. The disturbed process of angiogenesis which relies on a variety of signaling pathways may play a major role, since it has been shown that elevated Angiopoietin-2 (Ang-2) with declined Angiopoietin-1 (Ang-1) expression (Cui et al., 2011; Ye et al., 2011) and decreased vascular endothelial growth factor (VEGF) with increased angiostatin signaling (Zhu et al., 2010) are involved in dysfunctional cerebral neovascularization in diabetes. Among angiogenic regulators, accumulating data has led to the conclusion that Notch signaling plays a pivotal role in the control of vascular morphogenesis during development and in tumor angiogenesis (Phng and Gerhardt, 2009). A recent study has shown the implication of Jagged1-Notch1 pathway in stroke-induced angiogenesis (Ren et al., 2018). Specifically, another study revealed that Notch signaling was affected by diabetes mellitus associated with the retinal capillary regression, a potentially novel mechanism of diabetes-induced microvasculopathy at an early phase (Yoon et al., 2016). Hence, we suspect that Notch signaling pathway may play a part in the specific pathology where diabetes aggravates ischemic brain injury.

There exists a surplus of animal models in the study of T2DM, for example, genetic models namely GK rat and db/db mouse which develop spontaneous T2DM and show features resembling human pathology. The general development of T2DM in them is principally determined by gene unlike in humans, of which the etiology is characterized by both genetic background and multiple environmental components. Furthermore, the observations obtained from these genetically homogenous strains may not always be extended to the human population because of the large heterogeneity in the latter (Srinivasan et al., 2005). Therefore, our study focused on a suitable animal model with a combination of high fat diet (HFD) and low dose of streptozotocin (STZ) to not only share similar metabolic characteristics but mimic the natural development of human T2DM.

Building upon the above discoveries, our research sought to answer the following issues: (1) Are the cerebral microvasculopathy observed in the GK rat and db/db mice present in HFD/STZ-treated model of T2DM rats; (2) whether the Jagged1-Notch1 pathway is involved in the impaired baseline cerebral neovascularization and angiogenesis both before and after diabetic cerebral ischemia, by which brain injury is aggravated.

## Materials and Methods

### Animal

Male Sprague-Dawley rats (160 ± 10 g) were obtained from Shanghai JieSiJie Laboratory Animal Co., Ltd. and housed in Fudan University animal care facility, with approvement from our University Animal Care and Use Committee. All experiments followed the National Institute of Health guidelines for care and use of animals in research and were under protocols approved by the Committee on the Ethics of Animal Experiments of our university. All rats were randomly assigned to six groups: non-diabetic control (NC) group (*n* = 9), diabetic control (DC) group (*n* = 12), non-diabetic sham (NS) group (*n* = 9), diabetic sham (DS) group (*n* = 9), non-diabetic cerebral ischemic (NDI) group (*n* = 24), and diabetic cerebral ischemic (DI) group (*n* = 24). The rats were sacrificed on days 1, 3, 5, and 7 after cerebral ischemia.


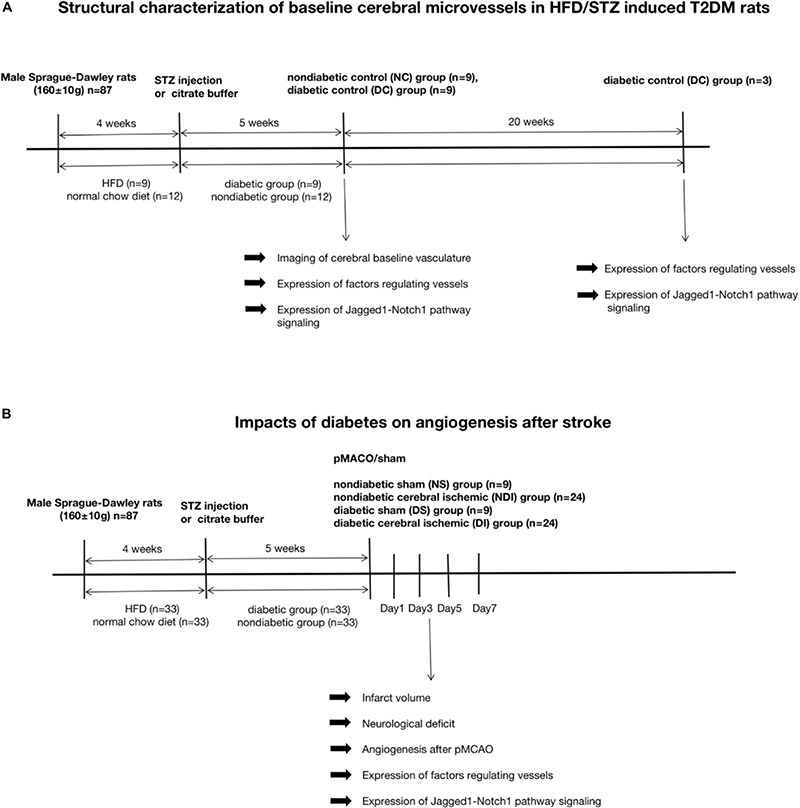


### T2DM Model

Since the combination of HFD-fed and low-dose STZ-treated rat serves as an alternative animal model for T2DM (Reed et al., 2000; Srinivasan et al., 2005; Gheibi et al., 2017), in our study, diabetes mellitus was induced in 4-week HFD (Slacom, China; 40% calories as fat) feeding male rats by injection of 35 mg/kg STZ (Sigma, United States) intraperitoneally. Control animals were fed with normal chow diet for 4 weeks, and received only citrate buffer. 3 and 7 days after injection, the HFD rats were considered as T2DM when their random blood glucose (RBG) levels exceeded 300 mg/dL. The RBG level of each rat was measured once a week after STZ injection using an ACCU-CHEK Performa blood glucose meter (Roche, Germany) until euthanasia.

The levels of serum lipids profile including total cholesterol (TC) [TC assay kit (A111-1-1, Jiancheng, China)], high-density lipoprotein cholesterol (HDL-C) [HDL-C assay kit (A112-1-1, Jiancheng, China)], low-density lipoprotein cholesterol (LDL-C) [LDL-C assay kit (A113-1-1, Jiancheng, China)], and triglycerides (TG) [TG assay kit (A110-1-1, Jiancheng, China)] were tested after euthanasia using commercially available colorimetric quantitation kits.

### Permanent Middle Cerebral Artery Occlusion (pMCAO) Model

After 5 weeks of diabetes induction, cerebral ischemia was induced by suture pMCAO as was described (Tu et al., 2019). Briefly, animals were anesthetized with an injection of 10% chloral hydrate (0.35 ml/100 g) intraperitoneally. Following exposure of the surgical field, the right common carotid artery (CCA), external carotid artery (ECA), and internal carotid artery (ICA) were isolated from the vagus nerve and these arteries were ligated temporarily using a cotton thread. The suture (Cinontech Co., China) was introduced into ICA and advanced until the tip occluded the middle cerebral artery (MCA), resulting in a cessation of blood flow. Laser Doppler flowmetry (Perimed, Sweden) monitoring the CBF value dynamically was used to confirm occlusion.

### Behavioral Assessment

Sensorimotor test, Garcia test, was performed (Garcia et al., 1995) in a blinded manner on days 1, 3, 5, and 7 after cerebral ischemia. The Garcia test, a composite neurological test used to evaluate various sensorimotor deficits, includes 6 examinations: spontaneous activity, symmetry in the movement of four limbs, forepaw outstretching, climbing, body proprioception, and response to vibrissae touch (Garcia et al., 1995). The score ranges between 3 and 18 and the lower the score, the worse the neurological function.

### Measurement of the Volume of Cerebral Infarction and Brain Edema

On 3rd day post-pMCAO, the rats were sacrificed. To quantify experimental cerebral infarction and brain edema volume, cryostat-cuts of coronal brain sections (30 μm) every 360 μm from antiunion were performed, and 15 sections were stained with cresyl violet totally (Sangong, China) and an image analysis system ImageJ was used to evaluate the lesion. The injury volume was calculated as a percentage of the contralateral non-lesioned area in each section (Swanson et al., 1990).

### Imaging of Cerebral Baseline Vasculature

To visualize cerebral baseline cortex vessels as previously described (Prakash et al., 2012, 2013a), animals of NC/DC groups received injection of 500 μL of 50 mg/mL fluorescein isothiocyanate (FITC)-dextran (molecular weight 2,000,000; Sigma-Aldrich) via jugular vein after deeply anesthetized with chloral hydrate. After 10 min, brains were isolated and immersed in 4% paraformaldehyde (24 h) followed by 30% sucrose in phosphate-buffered saline (PBS). Z-stacked confocal three-dimensional images were collected from 100 μm thick sections using OLYMPUS FLUOVIEW FV3000 confocal microscope. Z-step was defined as 1.984 μm, image size 512 × 512 pixels, 20 × lens. The mean value of three separate cortical images from three 100 μm apart sections were calculated to reduce variability. Image stacks were imported into FIJI and reconstructed three dimensionally. Vascular density is defined as the density of FITC-stained vasculature from the merged planes over the total section (Prakash et al., 2012).

### Angiogenesis After pMCAO

Angiogenesis detected by double immunolabeling of anti-CD31 [endothelial cells (ECs)] and anti-Ki67 (proliferating cells) was evaluated on days 1, 3, 5, and 7 after cerebral ischemia. Coronal 10-μm-thick sections were incubated with primary antibody (1:200, Abcam, United Kingdom) overnight at 4°C followed by appropriate Alexa Fluor 594 or 488–labeled secondary antibodies (1:1,000, Yishan, China) for 1 h at room temperature. Cell counts of the average number of Ki67^+^ and CD31^+^ cells in three regions of interest (ROI) located in the peri-infarct area were performed at three coronal brain levels (+ 0.80, -0.80, and -1.20 mm relative to bregma) that consistently contained the infarct area (Poittevin et al., 2015).

### Western Blot

The ischemic penumbra was collected as described previously (Ashwal et al., 1998). Equally 40 μg of protein was loaded in each well and resolved by SDS-PAGE electrophoresis. The membranes were immunoblotted with primary antibody: Ang-2 (1:5,000, Abcam, United Kingdom), VEGF-A (1:100, Santa Cruz, United States), Jagged1 (1:100, Santa Cruz, United States), Notch1 (1:1,000, CST, United States), NICD (1:1000, CST, United States), Hes1 (1:1,000, Abcam, United Kingdom), β-actin (1:1,000, Yishan, China) overnight at 4°C, then incubated with appropriate secondary antibody (1:4,000, Yishan, China) and finally visualized with an ECL kit (Epizyme, China).

### RNA Isolation and qRT-PCR

The total RNA was extracted by Trizol Reagent (Sigma, United States) and reverse transcription was performed with a cDNA Synthesis Kit (Takara, Japan). The resultant cDNAs were amplified with SYBR-Green Master Kits (Yeasen, China). The primers were as follows:

Hif-1a:  forward: 5′-TTCTCCAAGCCCTCCGAGTG
  TG-3′
                 reverse: 5′-GCGGTGGCAGTGACAGTGA     
                   TG-3′
VEGF-A:  forward: 5′-TACTGCTGTACCTCCACCATG     
                   CC-3′
                 reverse: 5′-GCAATAGCTGCGCTGGTAGA     
                   CG-3′
Ang-2:   forward: 5′-TCCAGACTGACGCACATCAC-3′
                 reverse: 5′-ATTTCTCCAGACCCGCAGTG-3′
Ang-1:   forward: 5′-TTCTTCGCTGCCATTCTGACTC     
                   AC-3′
                 reverse: 5′-CGCACTCTCACGGCAGTTCC-3′
PDGF-β:  forward: 5′-CTTGTTCTGGGACGCACTCTT     
                   GG-3′
                 reverse: 5′-GCTTCTCACTGCTTCTGGCTGT     
                   AG-3′
TGF-β:     forward: 5′-GCAACAATTCCTGGCGTTACC     
                   TTG-3′
                 reverse: 5′-TGTATTCCGTCTCCTTGGTTCA     
                   GC-3′
Jagged1:  forward: 5′- GAGCCCAACCCTTGCCAGA     
                   ATG-3′,
                 reverse: 5′-AGTTCTTGCCCTCGTAGTCCTC     
                   AG-3′
Notch1:  forward: 5′-TGCCGAGTGTGAGTGGGAT     
                   GG-3′
                 reverse: 5′-AAGTGGAAGGAGTTGTTGCGTA     
                   GC-3′
Hes1:   forward: 5′-TCCTGACGGCCAATTTGCTTT     
                   CC-3′
                 reverse : 5′-CTGGAAGGCGACACTGCGTT     
                   AG-3′
Hes5:   forward: 5′-GACCGCATCAACAGCAGCAT     
                   TG-3′
                 reverse: 5′-TCTCCAGGATGTCGGCCTTC     
                   TC-3′

### Immunofluorescence Staining

These procedures were performed as previously described (Tu et al., 2019). Serial 10 μm-thick coronal sections of the rat brain after fixation and dehydration were immersed in primary antibodies: Jagged1 (1:100, Santa Cruz, United States), Notch1 (1:1,000, CST, United States), Hes1 (1:100, Santa Cruz, United States), and CD31 (1:200, Abcam, United Kingdom)/CD31 (1:200, Affinity Biosciences, OH, United States) overnight at 4°C, followed by fluorescein-conjugated secondary antibodies (1:1,000, Yishan, China), and observed with fluorescence microscope (Olympus, Japan).

### Statistics

SPSS 25 was employed. Data were presented as mean ± standard error of the mean (SEM) if they conformed to normal distribution, and median ± IQR if not. *P* < 0.05 was considered statistically significant. Differences between groups was determined by a one-way ANOVA followed by Tukey test.

## Results

### General Conditions, Body Weight, Biochemical Analysis, and Mortality Rate

**FIGURE 1:**
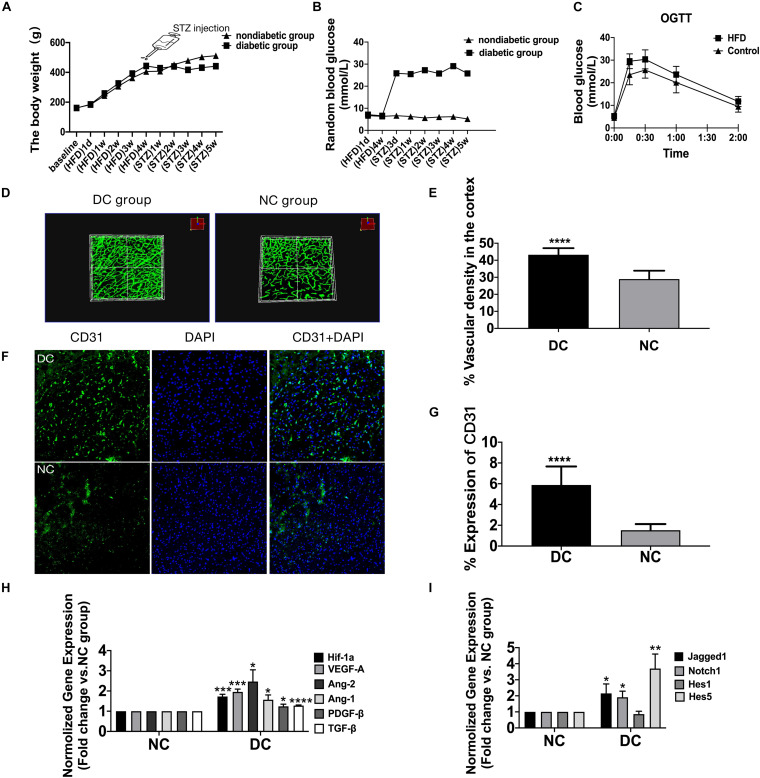
The enhanced yet dysfunctional baseline neovascularization in HFD/STZ rat model before cerebral ischemia. **(A)** Mean body weight changes during the time of high-fat diet and after the STZ treatment (*n* = 42 in each group). **(B)** Mean plasma glucose concentration changes during the time of high-fat diet and after the STZ treatment (*n* = 42 in each group). **(C)** Mean plasma glucose concentration in response to an oral glucose challenge in chow-fed and HFD-fed rats (*n* = 42 in each group). **(D)** Representative FITC-perfused cerebrovascular images from non-diabetic control and diabetic control rats showing differences in neovascularization in the cerebral cortex (Z-step was defined as 1.984 μm, image size 512 × 512 pixels, 20 × lens). **(E)** Significant differences in vascular density were observed in the cortex in both non-diabetic control and diabetic control groups. **(F)** The staining of ECs in the cerebral cortex of diabetic control and non-diabetic control. Magnification ×200. Scale bar = 100 μm. **(G)** ECs were markedly increased in the cortex of the diabetic control group compared with non-diabetic control group. **(H)** The gene expression of Hif-1a, VEGF-A, Ang-2, Ang-1, TGF-β, and PDGF-β was increased in diabetic control group compared with non-diabetic control group. **(I)** The gene expression of Jagged1, Notch1, Hes1, and Hes5 was increased in diabetic control group compared with non-diabetic control group. *p < 0.05 vs. NC group, **p < 0.01 vs. NC group, ***p < 0.001 vs. NC group, ****p < 0.0001 vs. NC group.

During the first 4 weeks of HFD, the rats fed the HFD weighed significantly heavier than rats fed the normal chow diet (*p* < 0.05), while the serum glucose concentrations of the two groups were similar (*p* > 0.05) (Table 1). Oral glucose tolerance test (OGTT) after 4-week HFD (Figure 1C) showed elevated serum glucose concentrations of HFD-fed rats compared with chow-fed rats (*p* < 0.05). After 4 weeks of dietary manipulation, injection of STZ significantly (*p* < 0.0001) increased serum glucose concentrations in HFD rats (Figure 1B and Table 1), which displayed the symptoms of polyuria, polydipsia, and polyphagia as compared to chow-fed control rats. In addition, STZ produced reduction in the body weights of the HFD-fed rats, which were still slightly higher than chow-fed rats on week 1 after injection and were significantly lower than chow-fed rats on week 3 after injection (*p* < 0.05) (Figure 1A and Table 1).

**TABLE 1:** The body weight/random blood glucose/serum lipid level in different groups.

		Non-diabetic group	Diabetic group
	Baseline	161.75 ± 2.58	161.13 ± 0.83
	(HFD)D1	183.88 ± 1.22	185.30 ± 1.33
	(HFD)W1	243.88 ± 3.73	258.70 ± 2.62*
	(HFD)W2	306.50 ± 6.24	326.06 ± 4.20*
Body weight (g)	(HFD)W2	363.00 ± 8.00	392.45 ± 5.77*
	(HFD)W4	405.88 ± 7.83	443.03 ± 7.04*
	(STZ)W1	408.13 ± 9.67	428.70 ± 8.30
	(STZ)W2	452.00 ± 10.85	440.04 ± 9.53
	(STZ)W3	479.50 ± 9.36	416.74 ± 9.90*^#^
	(STZ)W4	503.50 ± 10.37	431.50 ± 10.30**
	(STZ)W5	512.00 ± 18.88	441.37 ± 11.41*
	(HFD)D1	6.64 ± 0.14	7.12 ± 0.12
	(HFD)W4	6.36 ± 0.19	6.61 ± 0.17
	(STZ)D1	6.64 ± 0.14	25.86 ± 0.72****
Blood glucose (mmol/L)	(STZ)W1	6.27 ± 0.30	25.48 ± 1.16****
	(STZ)W2	5.60 ± 0.16	27.24 ± 1.68****
	(STZ)W3	6.06 ± 0.17	25.75 ± 1.66****
	(STZ)W4	6.19 ± 0.27	29.09 ± 1.35****
	(STZ)W5	5.19 ± 0.24	25.78 ± 1.77****
Serum lipid (mmol/L)	TC	1.87 ± 0.15	4.25 ± 0.45****
	TG	0.33 ± 0.06	0.78 ± 0.14**
	LDL-C	0.52 ± 0.07	1.68 ± 0.22****
	HDL-C	0.45 ± 0.07	0.20 ± 0.02**

*p < 0.05 vs. non-diabetic group, **p < 0.01 vs. non-diabetic group, ****p < 0.0001 vs. non-diabetic group, ^#^p < 0.05 vs. diabetic group (non-diabetic group n = 42, diabetic group n = 45).

The diabetic group also displayed higher levels of TC, LDL-C and TG and lower level of HDL-C than the non-diabetic group (*P* < 0.01) (Table 1).

The mortality rates were 7/52 in diabetic group, 0/9 in DS group, 5/29 in DI group, 0/9 in NS group, 4/28 in NDI group.

### The Enhanced Yet Dysfunctional Baseline Neovascularization in HFD/STZ Rat Model Before Cerebral Ischemia

**FIGURE 2:**
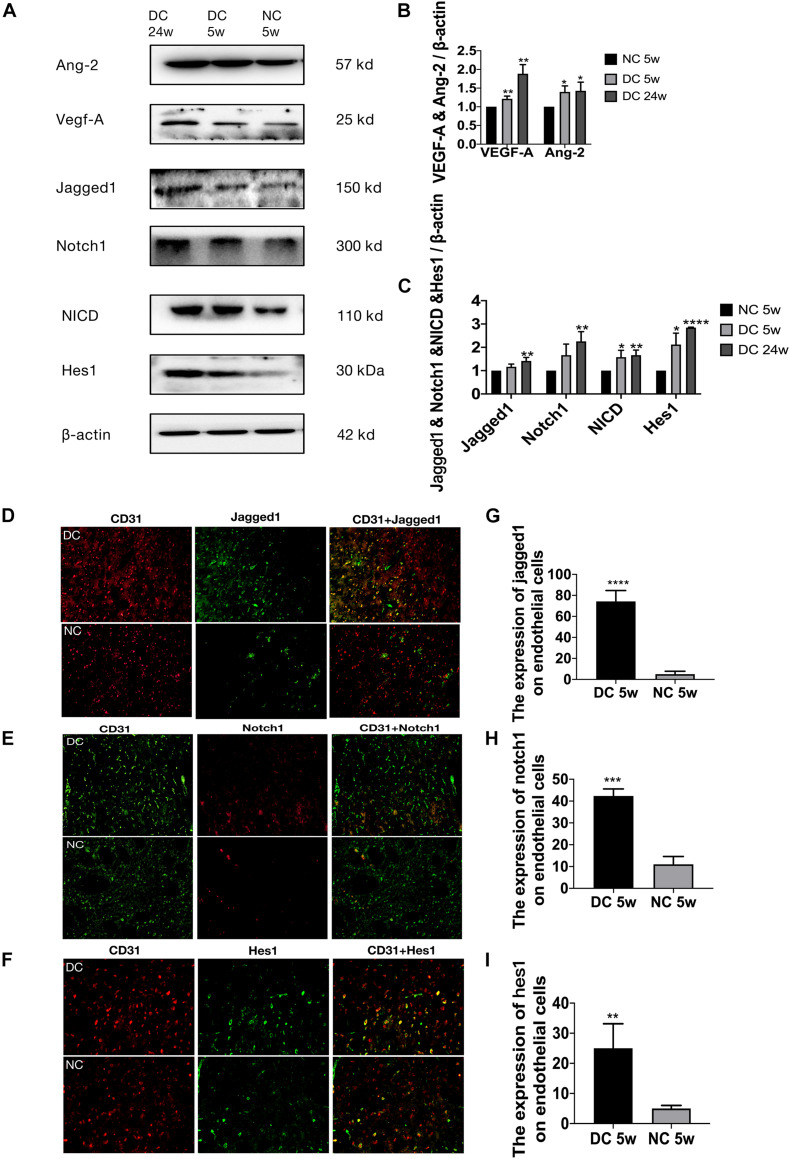
Altered Jagged1-Notch1 signaling with baseline neovascularization in T2DM. **(A–C)** The expression of VEGF-A, Ang-2, Jagged1, Notch1, NICD, and Hes1 in the cortex was assessed by Western blot (*n* = 3 per group). **(D–I)** The expression of Jagged1/Notch1/Hes1 and CD31 was assessed by immunofluorescence staining in the cortex (Magnification ×200. Scale bar = 100 μm) and the positive cells of Jagged1/Notch1/Hes1 and CD31 were calculated (*n* = 3 per group). *p < 0.05 vs. NC group, **p < 0.01 vs. NC group, ***p < 0.001 vs. NC group, ****p < 0.0001 vs. NC group.

In the diabetic group, the total cerebral microvasculature (Figure 1D) in the cortex was relatively greater than controls (*P* < 0.0001) (Figure 1E). In accordance with the FIJI data, there was also more CD31 staining (Figures 1F,G) (*P* < 0.0001), implying more non-productive newly formed microvessels. The VEGF-A detected ∼25 kDa (Figure 2A) was greater in the cortex of the diabetic group (*P* < 0.01) (Figure 2B). Moreover, the expression of VEGF-A in 24 w diabetic rats was much greater than 5 w diabetic rats, and similar changes were observed in the expression of Ang-2 (Figures 2A,B). Also, hypoxia-inducible factor-1 alpha (Hif-1a) mRNA, VEGF-A mRNA, and Ang-2 mRNA were significantly upregulated in the cortex in the diabetic group (Figure 1H). In addition, mRNAs for Ang-1, transforming growth factor-β (TGF-β), and platelet-derived growth factor-β (PDGF-β) were slightly increased in the diabetic group (Figure 1H).

### Altered Jagged1-Notch1 Signaling With Baseline Neovascularization in T2DM

Jagged1/Notch1/Hes1 was expressed on CD31-positive cells in the diabetic group but barely expressed on CD31-positive cells in the non-diabetic group (*P* < 0.01) (Figures 2D–I). In addition, we found increased expression of mRNA of Jagged1, Notch1, and Hes5 (Figure 1I) and protein of Jagged1, Notch1, NICD, and Hes1 (Figures 2A,C) in the diabetic group (*P* < 0.05).

### Larger Cerebral Infarction, Severer Brain Edema and Poorer Neurological Function in T2DM

**FIGURE 3:**
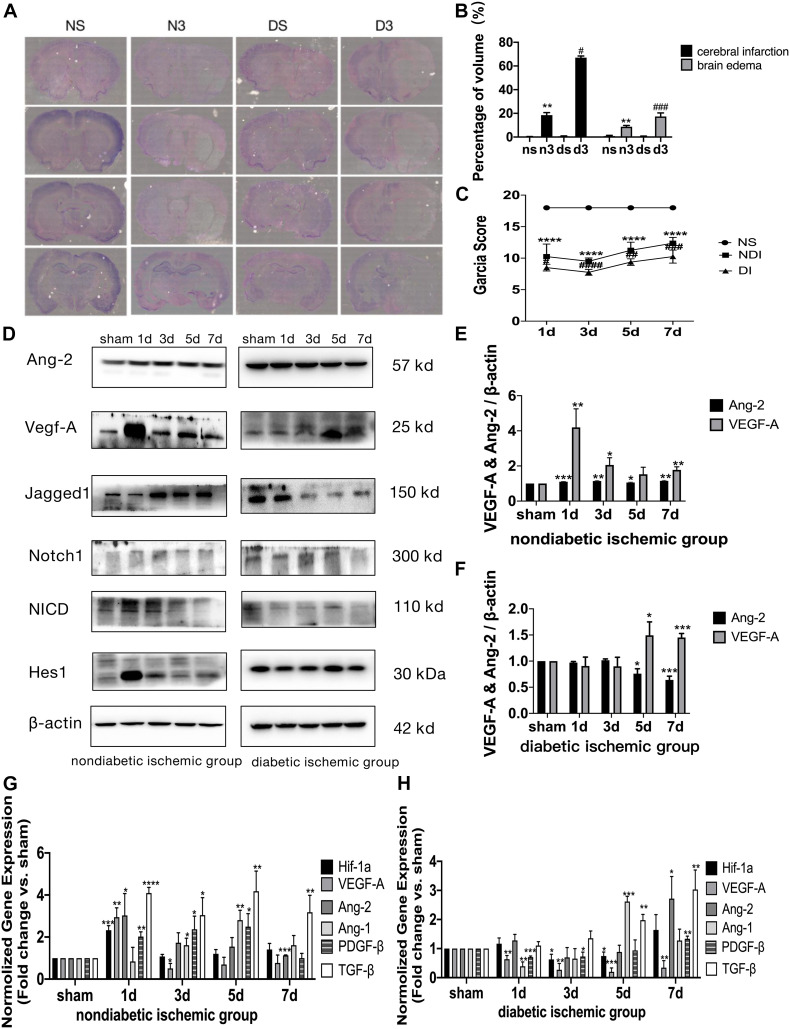
Delayed angiogenesis after acute ischemic stroke in T2DM. **(A)** Cresyl violet (CV) staining on day 3. NS, non-diabetic sham group (Day 3); N3, non-diabetic cerebral ischemic group (Day 3); DS, diabetic sham group (Day 3); D3, diabetic cerebral ischemic group (Day 3). The unstained area was the infarction area. **(B)** The volume of cerebral infarction and brain edema was calculated by CV staining on day 3. **(C)** Garcia score was evaluated on D1–D7 after cerebral ischemia (*n* = 8 per group). **(D)** The expression of VEGF-A, Ang-2, Jagged1, Notch1, NICD, and Hes1 in ischemic penumbra was assessed by Western blot (*n* = 3 per group). **(E,F)** The expression of VEGF-A and Ang-2 protein were calculated in NDI group and DI group. **(G,H)** The gene expression of Hif-1a, VEGF-A, Ang-2, Ang-1, TGF-β, and PDGF-β was showed in NDI group and DI group. *p < 0.05 vs. sham group, **p < 0.01 vs. sham group, ***p < 0.001 vs. sham group, ****p < 0.0001 vs. sham group; ^#^*p* < 0.05 vs. NDI group, ^##^*p* < 0.01 vs. NDI group, ^###^*p* < 0.001 vs. NDI group, ^####^*p* < 0.0001 vs. NDI group.

Compared with NS group, NDI group had larger cerebral infarction and severer brain edema (*p* < 0.01), while DI group had larger cerebral infarction (*p* < 0.05) and brain edema (*p* < 0.001) than NDI group (Figures 3A,B). In the Garcia test, NDI group scored significantly lower than NS group from days 1 to 7 (*p* < 0.0001), while DI group scored significantly lower than NDI group (*p* < 0.05) (Figure 3C).

### Delayed Angiogenesis After Acute Ischemic Stroke in T2DM

**FIGURE 4:**
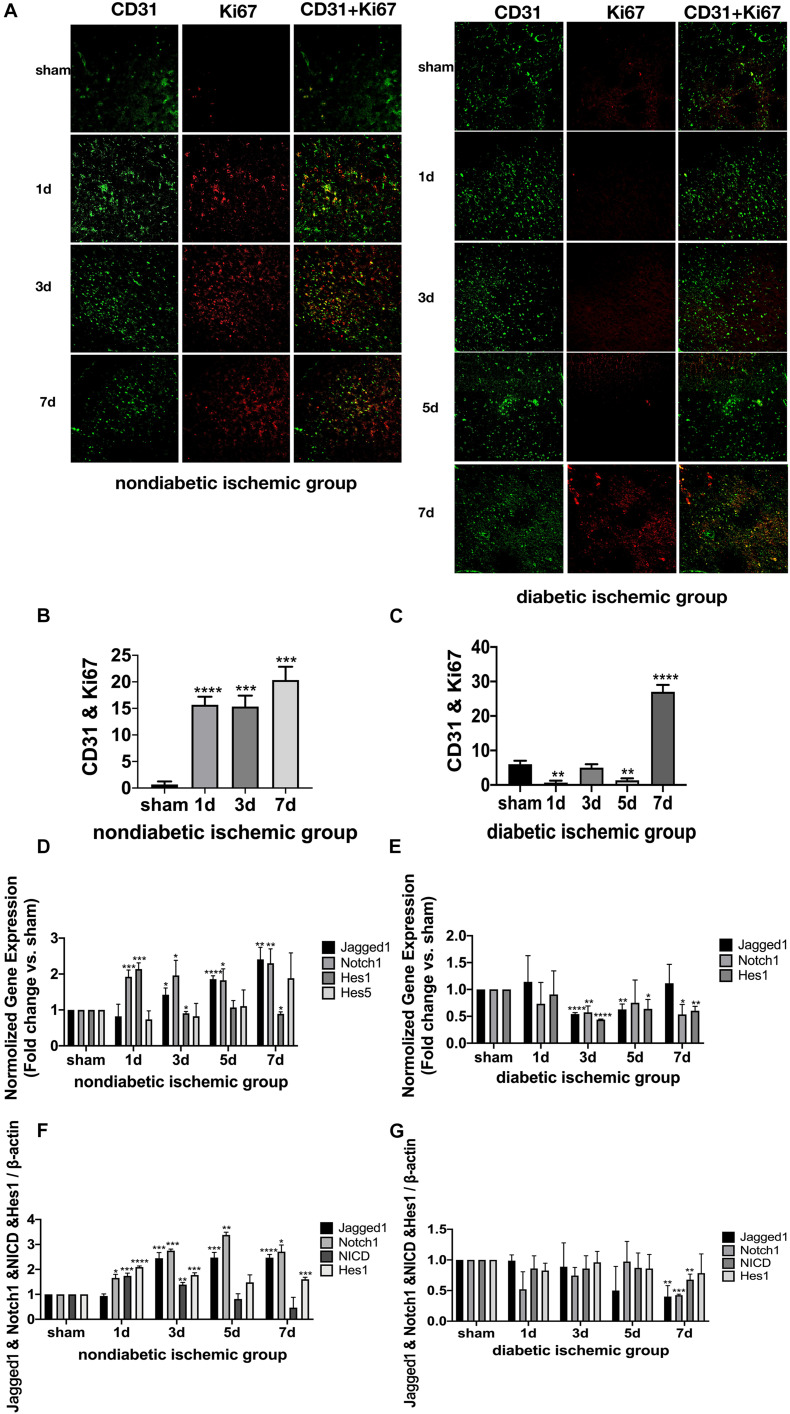
Suppressed Jagged1-Notch1 signaling with hampered angiogenesis after acute ischemic stroke in T2DM. **(A–C)** The expression of Ki67 and CD31 was assessed by immunofluorescence staining in ischemic penumbra (Magnification * 200. Scale bar = 100 μm) and the positive cells of Ki67 and CD31 were calculated (*n* = 3 per group). **(D,E)** The gene expression of Jagged1, Notch1, Hes1, and Hes5 was showed in NDI and DI group. **(F,G)** The expression of Jagged1, Notch1, NICD, and Hes1 protein were calculated in NDI group and DI group. *p < 0.05 vs. sham group, **p < 0.01 vs. sham group, ***p < 0.001 vs. sham group, ****p < 0.0001 vs. sham group.

At D1 after ischemic stroke, EC proliferation was significantly detected in NDI group, while there wasn’t a significant increase until D7 in DI group (Figures 4A–C). Meanwhile, Ang-2, Ang-1, TGF-β, and PDGF-β mRNA levels weren’t increased until D7 in DI group compared with DS group (Figure 3H). In contrast, at D1 after ischemic stroke, Hif-1a, VEGF-A, Ang-2, TGF-β, and PDGF-β mRNA were significantly increased in NDI group compared with NS group (Figure 3G). Similar changes exist in the protein expression of VEGF-A and Ang-2 in two groups (Figures 3D–F).

### Suppressed Jagged1-Notch1 Signaling With Hampered Angiogenesis After Acute Ischemic Stroke in T2DM

**FIGURE 5:**
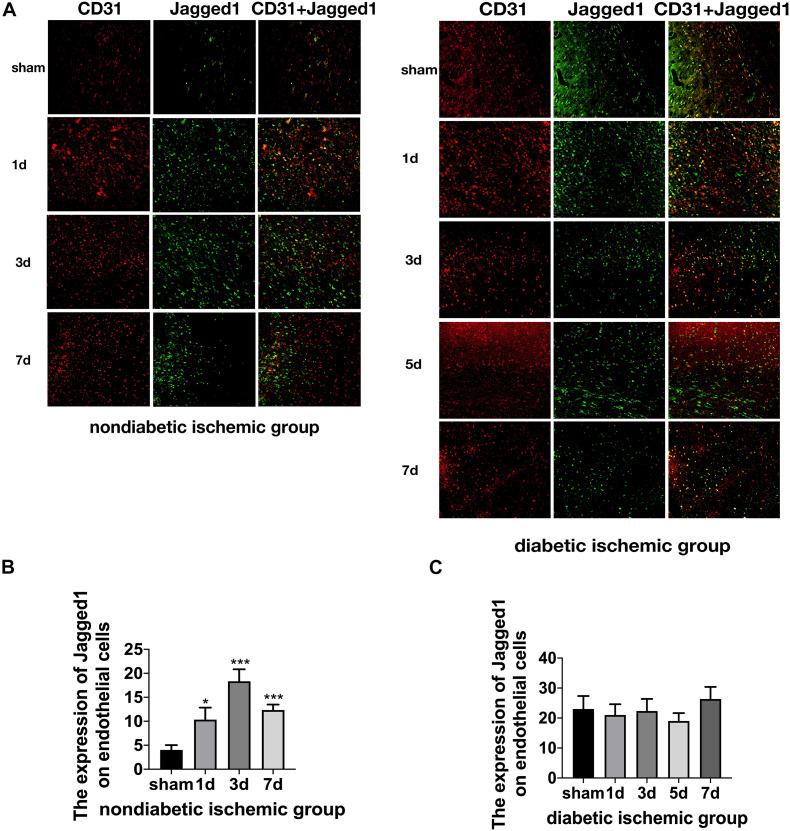
Suppressed Jagged1-Notch1 signaling after acute ischemic stroke in T2DM. **(A–C)** The expression of Jagged1 and CD31 was assessed by immunofluorescence staining in ischemic penumbra (Magnification ×200. Scale bar = 100 μm) and the positive cells of Jagged1 and CD31 were calculated (*n* = 3 per group). *p < 0.05 vs. sham group, ***p < 0.001 vs. sham group.

**FIGURE 6:**
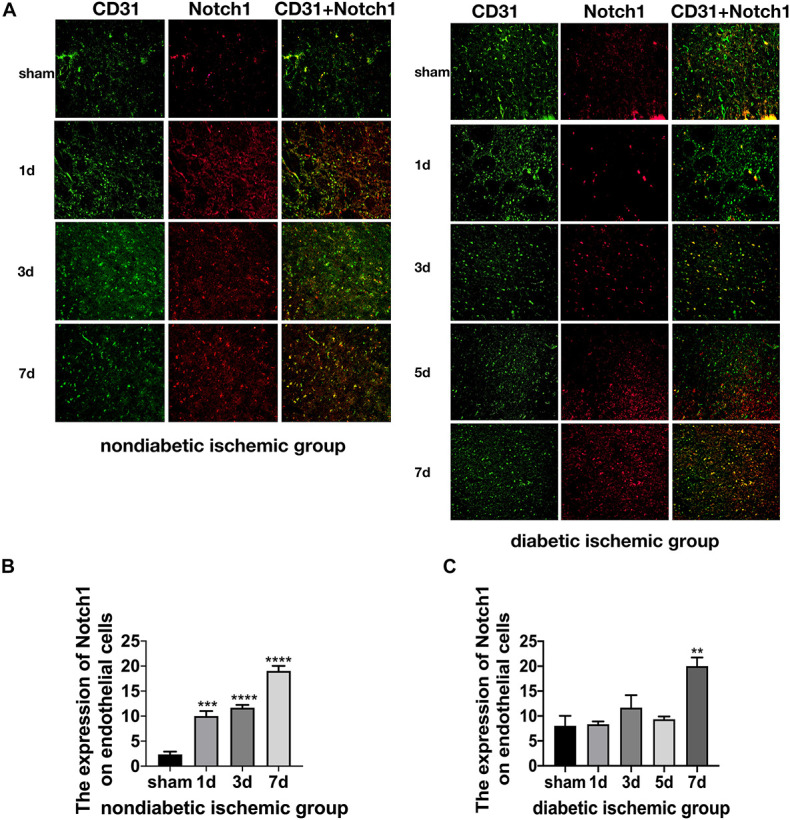
Suppressed Jagged1-Notch1 signaling after acute ischemic stroke in T2DM. **(A–C)** The expression of Notch1 and CD31 was assessed by immunofluorescence staining in ischemic penumbra (Magnification ×200. Scale bar = 100 μm) and the positive cells of Notch1 and CD31 were calculated (*n* = 3 per group). **p < 0.01 vs. sham group, ***p < 0.001 vs. sham group, ****p < 0.0001 vs. sham group.

**FIGURE 7:**
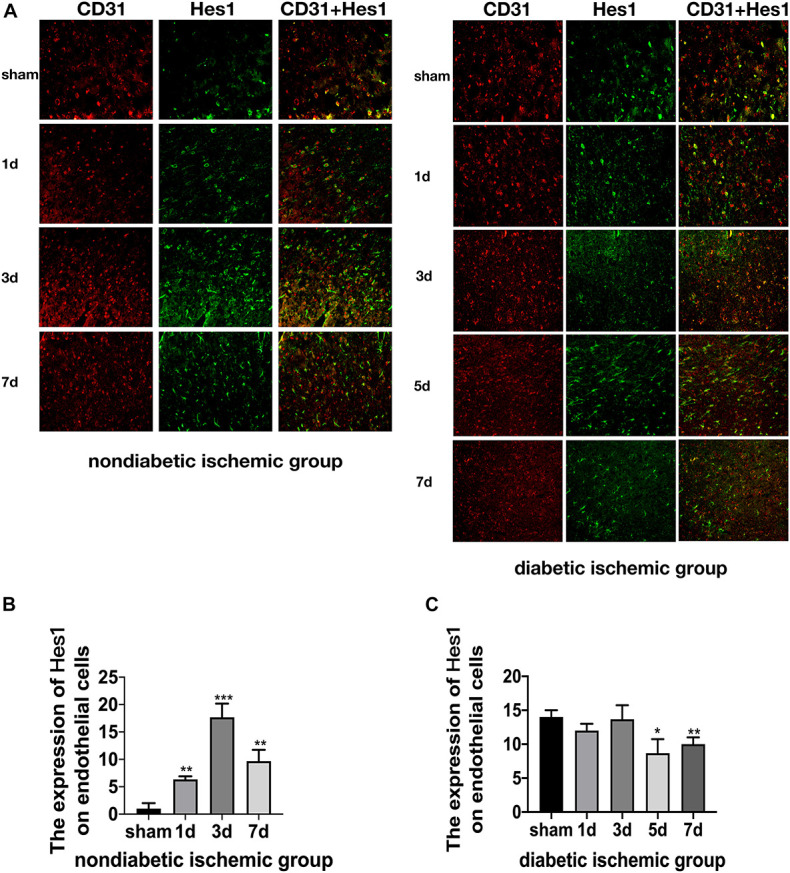
Suppressed Jagged1-Notch1 signaling after acute ischemic stroke in T2DM. **(A–C)** The expression of Hes1 and CD31 was assessed by immunofluorescence staining in ischemic penumbra (Magnification ×200. Scale bar = 100 μm) and the positive cells of Hes1 and CD31 were calculated (*n* = 3 per group). *p < 0.05 vs. sham group, **p < 0.01 vs. sham group, ***p < 0.001 vs. sham group.

The expression of Jagged1 and Notch 1 was significantly upregulated on ECS in the peri-infarct region of NDI group at D1 than the sham group (Figures 5A, 6A) (*P* < 0.05). Moreover, Hes1, a downstream target of Notch1, was expressed on ECS in NDI group at D1, but rarely detected in the sham group (Figure 7A) (*P* < 0.01). However, the expression of Jagged1, Notch1, and Hes1 on ECS failed to elevate in DI group compared with DS group (Figures 5–7). In addition, qPCR (Figures 4D,E) and western blot (Figure 3D) revealed that after ischemic stroke, the expression of Notch1, Jagged1, and Hes1 mRNA (Figure 4D) and Notch1, Jagged1, NICD, and HES1 protein (Figures 3D, 4F) was significantly increased at D1 and D3 in NDI group. In contrast, the expression of the Jagged1-Notch1 signaling pathway was not upregulated in diabetic group (Figures 3D, 4E,G).

## Discussion

In the present study, we provided novel evidence of increased yet dysfunctional baseline neovascularization in the cerebrovasculature of HFD/STZ rats accompanying with the activated Jagged1-Notch1 signaling. Furthermore, we observed delayed angiogenesis after cerebral ischemia as well as suppressed Jagged1-Notch1 signaling in T2DM rats compared with non-diabetic rats.

The impact of diabetes on cerebral vasculature has not been investigated until recent years (Li et al., 2010; Prakash et al., 2012, 2013a) and various models of diabetes have been utilized. There is enhanced arteriogenesis and angiogenesis in the brain of GK rats (Li et al., 2010). Similar changes were observed in another model of T2DM, db/db mice (Prakash et al., 2013a). Our study focused on a unique T2DM model—HFD/STZ rats, an animal model not predominantly genetically determined. This model combines HFD and pancreatic β-cell toxin STZ (Reed et al., 2000), making it a more suitable animal model because it can not only mimic the natural history of T2DM but include the large heterogeneity which commonly exists in human population. We found that in HFD/STZ rats, there was enhanced baseline neovascularization in the cerebrovasculature, which is similar to other reports (Li et al., 2010; Prakash et al., 2012, 2013a). The fact that these animal models of T2DM are made from various strains with different disease duration evidently indicates that T2DM has a broad impact on the microvasculature of the brain.

Our study examined the expression of Hif-1a and VEGF-A in the cortex of diabetic group and non-diabetic controls. While physiologic angiogenesis depends on a tightly coordinated balance between considerable anti-angiogenic and pro-angiogenic factors, it is well recognized that VEGF-A plays a pivotal role in this equilibrium (Ferrara, 2004). Substantial evidence also implicates VEGF as a mediator of pathological angiogenesis in diabetic retinopathy (Nakagawa et al., 2009; Wang et al., 2009; Advani and Gilbert, 2012). Furtherly, Hif-1 has been proposed to be a major stimulus to the activation of VEGF gene transcription (Forsythe et al., 1996). In our study, we found increased levels of both Hif-1a and VEGF-A in diabetes, which may imply that in diabetes early vascular dysfunction and decreased blood flow (Kelly-Cobbs et al., 2012) generate a hypoxic milieu that may be the initial incentive for enhanced cerebral microvasculature.

Except for Hif-1a/VEGF levels, an intact neovascularization process is controlled by the delicate balance between proangiogenic growth factors (VEGF-A and Ang-2) and stabilization and maturation factors (TGF-β, Ang-1, and PDGF-β) which is crucial to the blood vessel stabilization. The increased ratio of Ang-2/Ang-1 was found to be related to pro-angiogenic activity (Watanabe et al., 2005) and immature nature of vasculature (Pfister et al., 2010) in diabetic retinopathy. Meanwhile, increased Ang-2 but reduced Ang-1 expression may contribute to cerebral vascular damage after stroke in T2DM mice (Cui et al., 2011). In our study, we have shown that prior to cerebral ischemia, VEGF-A mRNA and Ang-2 mRNA were significantly increased in the cortex in the diabetic group compared with the non-diabetic group, while mRNAs for Ang-1, TGF-β, and PDGF-β were slightly increased, which may suggest the brain microvasculature in diabetic rat is immature. This is in conformity with another model of T2DM, the GK rat, in which augmented cerebral microvasculature was related to poor vascular mural maturity as revealed by less pericyte support and more non-perfused new vessel formation (Prakash et al., 2012). Interestingly, increased cerebral microvasculature of a mice model of type 1 diabetes with a longer duration is immature with a decrease in VEGF-A, Ang-1, PDGF-β, and TGF-β mRNA levels (Poittevin et al., 2015), which is in contrast to our findings. In our study, increased yet immature microvessels was accompanied by the upregulation of VEGF-A and Ang-2 mRNA and protein levels. Several factors including the different animal models of diabetes, the specific strains, and the severity of diabetes may explain the distinctions between the two studies.

During acute ischemic stroke, insufficient blood flow to the brain and the poor tissue oxygen tension often results in reparative neovascularization in order to attend the instant needs of brain high metabolism. It is evident that numerous proangiogenic growth factors are upregulated as early as hours after the onset of cerebral ischemia in rodents (Hayashi et al., 2003). In our study, we have shown that at D1 after cerebral ischemia, there was a significant upregulation of VEGF-A, Ang-2, TGF-β, and PDGF-β mRNA in NDI group compared with NS group. Moreover, it has been reported that poststroke triggered angiogenesis leads to increased vessel density from D1 to D21 (Hayashi et al., 2003). In the current study, EC proliferation was significantly observed in the NDI group from D1 to D7, which is also consistent with the above previous study. And we know that there was a significant correlation between the number of blood vessels in the peri-infarct regions and survival time (Krupinski et al., 1993, 1994), indicating that angiogenesis stimulation may have a beneficial effect on the damaged brain after ischemic stroke. However, we observed that diabetes impaired the recovery process by delaying angiogenesis after stroke, which is similar with the type 1 diabetes model (Poittevin et al., 2015). In our study, Ang-2, Ang-1, TGF-β, and PDGF-β mRNA levels were not increased until D7 in DI group compared with DS group. VEGF-A and Ang-2 protein followed the same time course. Furthermore, there wasn’t a significant increase of ECs until D7 in DI group, which is consistent with a previous study where while control animals displayed reparative neovascularization in both ischemic and non-ischemic hemispheres compared with the sham group, vascular density in the peri-infarct area was significantly reduced in diabetes mellitus (Prakash et al., 2013b). Collectively, impaired angiogenesis after cerebral ischemia in diabetic models may underlie why T2DM aggravates the ischemic brain injury.

The mechanisms of how diabetes leads to the dysfunctional neovascularization and impaired repair process after stroke are unclear and likely to contribute to assorted factors. Here we examined if the Jagged1-Notch1 pathway is involved in the impaired angiogenesis in diabetes. Accumulating evidences in vascular biosciences has shown that intrinsic signaling interactions between ECs play vital roles in the physiology and pathology of blood vessels (Potente et al., 2011). Furthermore, the movement of a single EC integrated into the vascular morphology is regulated by these intercellular signaling (Arima et al., 2011). Notch receptors and their ligands, delta-like ligand 4 (Dll4), and Jagged1, underlie the dynamic and transient regulation. Dll4-Notch1 signaling between neighboring ECs within the sprouting angiogenesis serves to restrict tip-cell formation in adjacent (stalk) ECs (Hellström et al., 2007). The vessel-stabilizing activity of the Dll4-Notch1 interaction is antagonized by Jagged1, which is proangiogenic and functions by downregulating Dll4-Notch1 signaling and leads to the excessive but immature vessel plexus (Benedito et al., 2009). In our study, numbers of Notch1^+^CD31^+^, Jagged1^+^CD31^+^ cells, and Hes1CD31^+^ cells were significantly increased in the diabetic group compared with the non-diabetic group. In addition, the mRNA of Jagged1, Notch1 and Hes5 and protein of Jagged1, Notch1, NICD, and Hes1 were increased in the diabetic group. Based on that, we have discovered that Jagged1-Notch1 signaling in adult rat was activated in diabetes mellitus associated with enhanced yet maybe immature cerebral neovascularization, a potentially novel mechanism of cerebral microvascular complications of diabetes. Intrudingly, when these diabetic rats were subjected to ischemic stroke, the Jagged1-Notch1 signaling pathway couldn’t be further stimulated by the ischemic event while it has been activated in the non-diabetic ischemic group (Ren et al., 2018). Our study has shown that after ischemic stroke the expression of Notch1, Jagged1 and HES1 on ECs at D1, D3, and D7 in NDI group was significantly increased, while in DI group, the expressions of Notch1, Jagged1, and Hes1 in the peri-infarct region were not increased where there was delayed angiogenesis at acute phase. Therefore, although the causality cannot be strictly verified, we highly suspect that the baseline cerebral microvasculopathy and activated Jagged1-Notch1 signaling in T2DM before ischemic stroke is at least partly responsible for the delayed angiogenesis as well as suppressed Jagged1-Notch1 signaling which cannot be further stimulated after pMCAO.

There are a few limitations in this study. Firstly, we chose to develop a chronic induced T2DM rat model in order to better mimic clinical pathology yet with confounding factors. However, since dyslipidemia is also responsible for vascular impairment (Zechariah et al., 2013), it might be difficult to attribute the microvascular impairment solely to hyperglycemia. Secondly, although HFD/STZ induction is a recognized model of T2DM, it cannot totally involve the complex pathophysiology observed in T2DM patients. Furthermore, whether there is a causal relationship between the impaired cerebral microvasculature and altered Jagged1-Notch1 signaling pathway needs to be further verified.

In conclusion, our study investigated the cerebral microvascular impairment before and after ischemic stroke in T2DM rats and the altered expression of Jagged1-Notch1 signaling pathway, a potentially novel mechanism of diabetes-related cerebral microvasculature dysfunction. Our findings may provide a basis for the development of novel treatment as management of diabetic stroke patients.

## Data Availability Statement

The datasets presented in this study can be found in online repositories. The names of the repository/repositories and accession number(s) can be found in the article/Supplementary Material.

## Ethics Statement

The animal study was reviewed and approved by the guidelines of animal care and use established by Shanghai Fudan University.

## Author Contributions

XQW, WF, and HFB conceived and designed the experiments. ZHG, JJ, YLT, CJ, CG, and FFS performed the experiments. ZHG and XQW wrote the manuscript. All authors confirm that they are the original contributors of this work and approved it for its submission.

## Conflict of Interest

The authors declare that the research was conducted in the absence of any commercial or financial relationships that could be construed as a potential conflict of interest.

## Abbreviations

T2DM: type 2 diabetes mellitus
pMCAO: permanent middle cerebral artery occlusion
ECs: endothelial cells
Hif-1a: hypoxia-inducible factor-1 alpha
VEGF-A: vascular endothelial growth factor A
Ang-2: Angiopoietin-2
Ang-1: Angiopoietin-1
PDGF- β: platelet derived growth factor beta
TGF- β: transforming growth factor beta
TG: Triglyceride
TC: Total cholesterol
LDL-C: low density lipoprotein cholesterol
HDL-C: high density lipoprotein cholesterol
STZ: streptozotocin
HFD: high fat diet
RBG: random blood glucose
OGTT: oral glucose tolerance test
NC: non-diabetic control
DC: diabetic control
NS: non-diabetic sham
NDI: non-diabetic cerebral ischemia
DS: diabetic sham
DI: diabetic cerebral ischemia
GK: Goto-Kakisaki Wistar rats
CCA: common carotid artery
ICA: internal carotid artery
ECA: external carotid artery
MCA: middle cerebral artery
CBF: cerebral blood flow
ROI: regions of interest.
