# Supplementary material for: Altered Jagged1-Notch1 Signaling in Enhanced Dysfunctional Neovascularization and Delayed Angiogenesis After Ischemic Stroke in HFD/STZ Induced Type 2 Diabetes Rats
Source: Front Physiol. 2021 Jul 8;12:687947. doi: 10.3389/fphys.2021.687947 (PMC8297620; doi:10.3389/fphys.2021.687947)
Supplement: Supplementary file 1 [file Presentation_1.zip › Supplementary Material Presentation.docx]

Supplementary Material

## Supplementary Figures


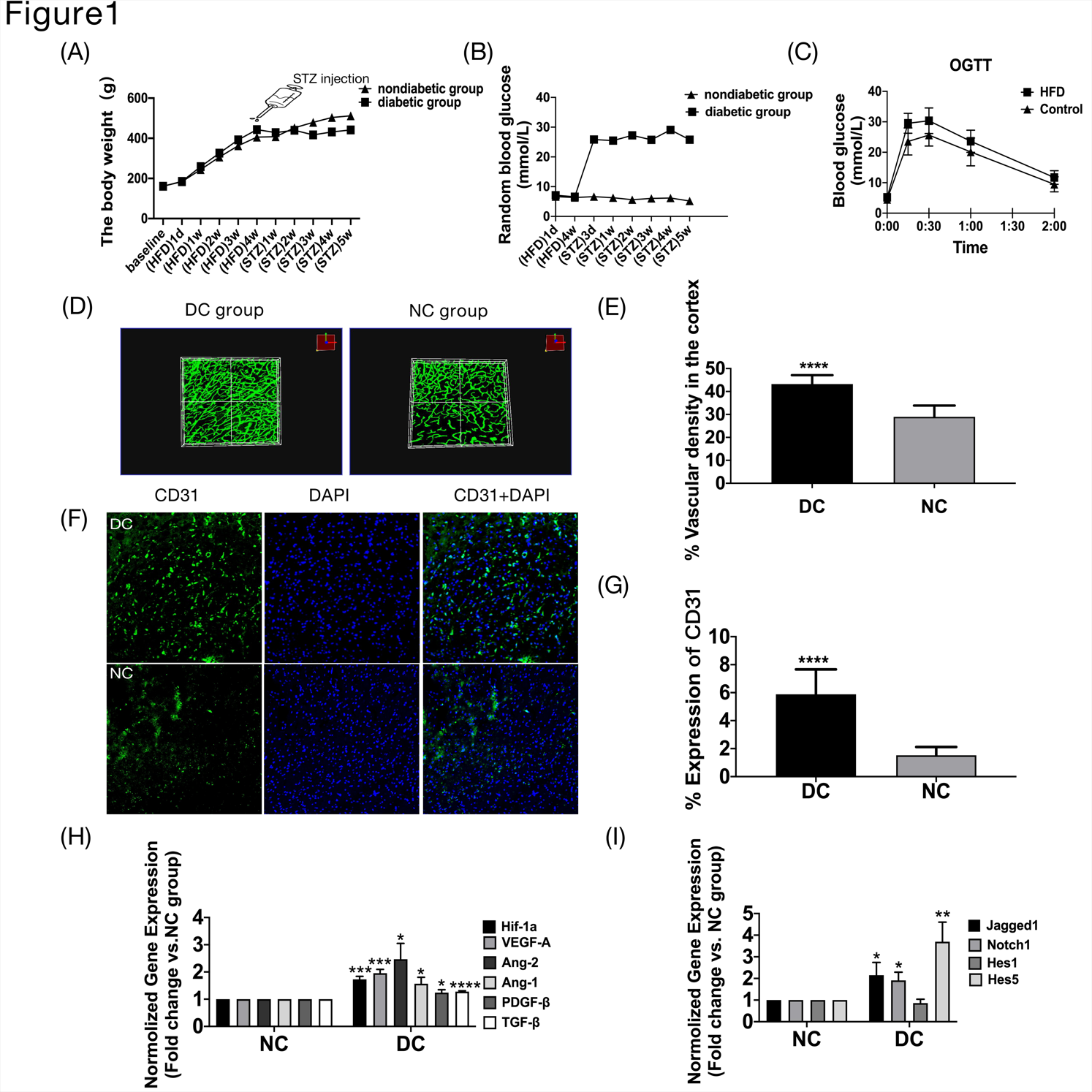


**Supplementary Figure 1.** The enhanced yet dysfunctional baseline neovascularization in HFD/STZ rat model before cerebral ischemia. (A) Mean body weight changes during the time of high-fat diet and after the STZ treatment (n=42 in each group). (B) Mean plasma glucose concentration changes during the time of high-fat diet and after the STZ treatment (n=42 in each group). (C) Mean plasma glucose concentration in response to an oral glucose challenge in chow-fed and HFD-fed rats (n=42 in each group). (D) Representative FITC-perfused cerebrovascular images from nondiabetic control and diabetic control rats showing differences in neovascularization in the cerebral cortex (Z-step was defined as 1.984 µm, image size 512 × 512 pixels, 20 × lens). (E) Significant differences in vascular density were observed in the cortex in both nondiabetic control and diabetic control groups. (F) The staining of ECs in the cerebral cortex of diabetic control and nondiabetic control. Magnification * 200. Scale bar = 100um. (G) ECs were markedly increased in the cortex of the diabetic control group compared with nondiabetic control group. (H) The gene expression of Hif-1a, VEGF-A, Ang-2, Ang-1, TGF-β, and PDGF-β was increased in diabetic control group compared with nondiabetic control group. (I) The gene expression of Jagged1, Notch1, Hes1 and Hes5 was increased in diabetic control group compared with nondiabetic control group. * p < 0.05 vs. NC group, ** p < 0.01 vs. NC group, *** p < 0.001 vs. NC group, **** p <0.0001 vs. NC group.


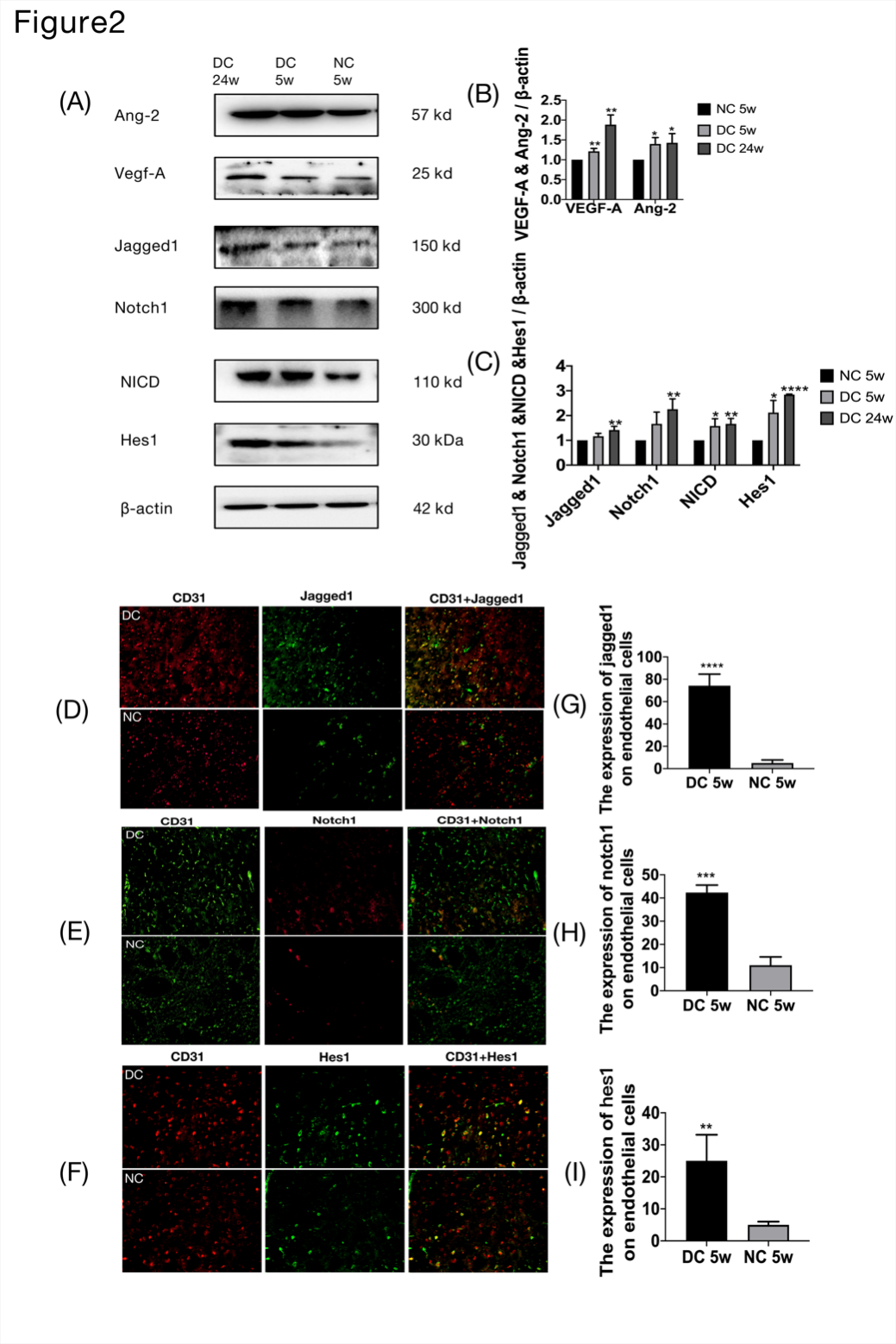


**Supplementary Figure 2.** Altered Jagged1-Notch1 signaling with increased baseline neovascularization in T2DM. (A-C) The expression of VEGF-A, Ang-2, Jagged1, Notch1, NICD and Hes1 in the cortex was assessed by Western blot (n = 3 per group). (D-I) The expression of Jagged1/Notch1/Hes1 and CD31 was assessed by immunofluorescence staining in the cortex (Magnification * 200. Scale bar = 100um) and the positive cells of Jagged1/Notch1/Hes1 & CD31 were calculated (n = 3 per group). * p < 0.05 vs. NC group, ** p < 0.01 vs. NC group, *** p < 0.001 vs. NC group, **** p <0.0001 vs. NC group.


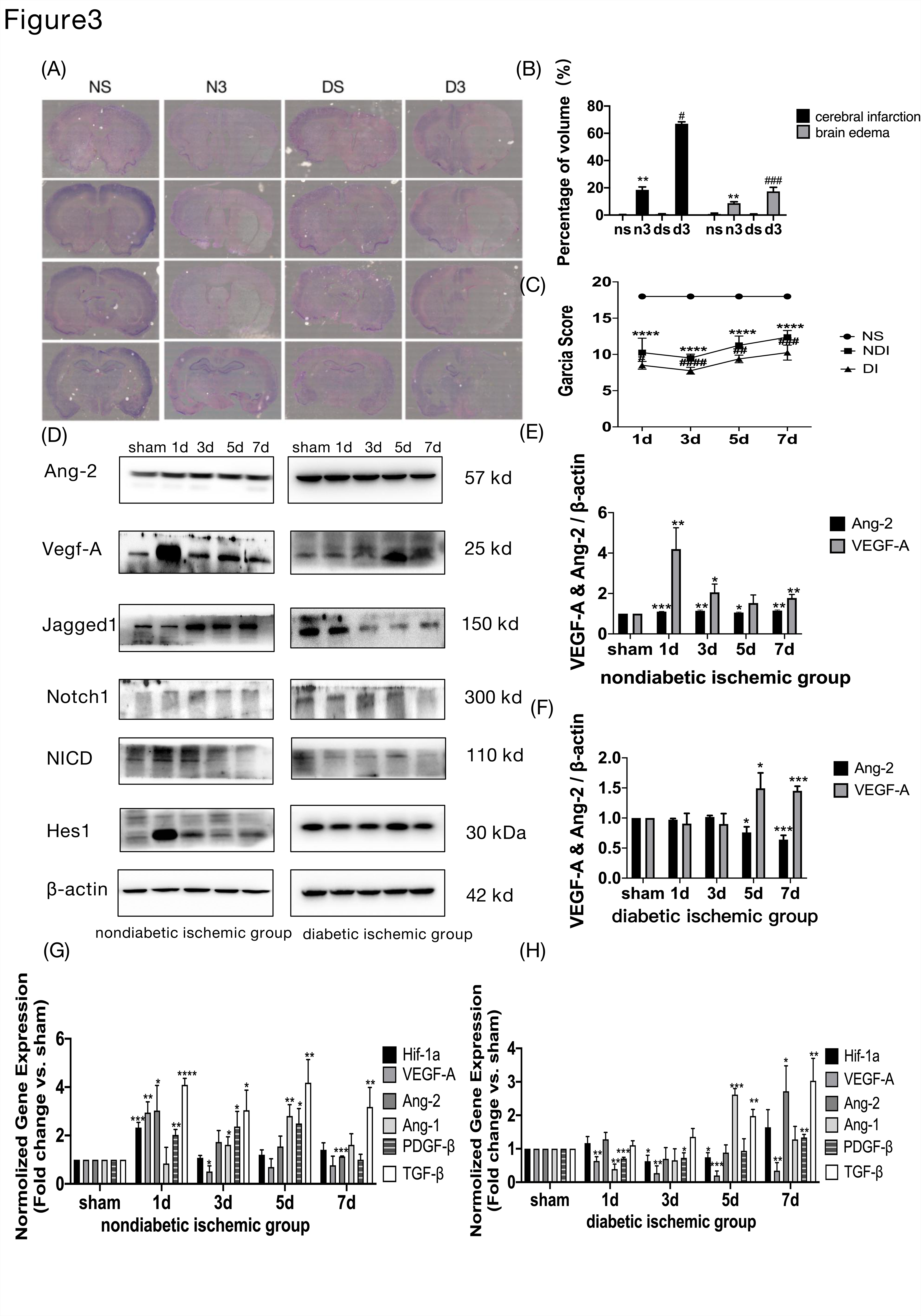


**Supplementary Figure 3.** Delayed angiogenesis after acute ischemic stroke in T2DM. (A) Cresyl violet (CV) staining on day 3. NS, nondiabetic sham group (Day3); N3, nondiabetic cerebral ischemic group (Day3); DS, diabetic sham group (Day3); D3, diabetic cerebral ischemic group (Day3). The unstained area was the infarction area. (B) The volume of cerebral infarction and brain edema was calculated by CV staining on day 3. (C) Garcia score was evaluated on D1 to D7 after cerebral ischemia (n = 8 per group). (D) The expression of VEGF-A, Ang-2, Jagged1, Notch1, NICD and Hes1 in ischemic penumbra was assessed by Western blot (n = 3 per group). (E-F) The expression of VEGF-A and Ang-2 protein were calculated in NDI group and DI group. (G-H) The gene expression of Hif-1a, VEGF-A, Ang-2, Ang-1, TGF-β, and PDGF-β was showed in NDI group and DI group. * p < 0.05 vs. sham group, ** p < 0.01 vs. sham group, *** p < 0.001 vs. sham group, **** p <0.0001 vs. sham group; # p < 0.05 vs. NDI group, ## p < 0.01 vs. NDI group, ### p < 0.001 vs. NDI group, #### p <0.0001 vs. NDI group.


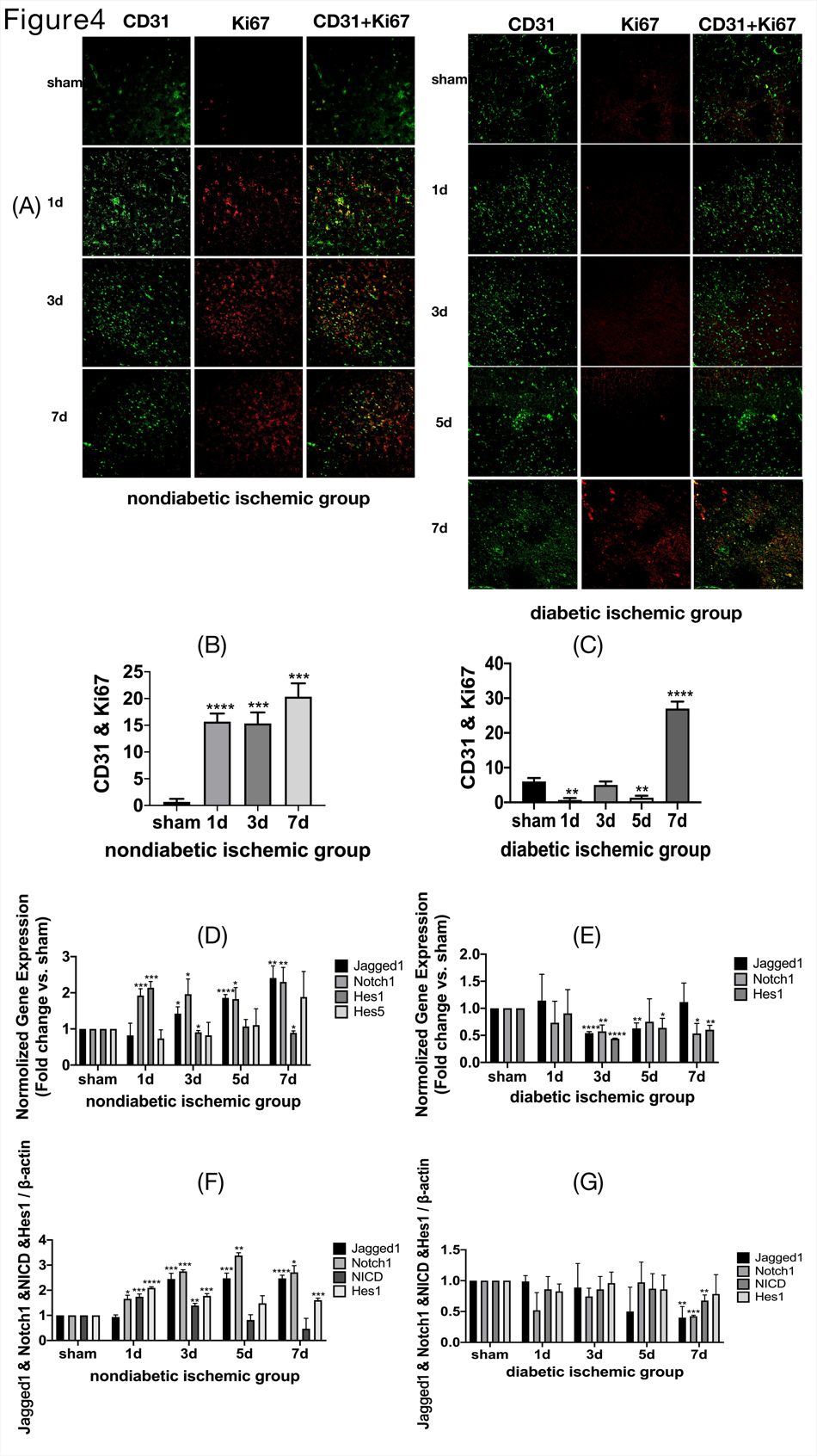


**Supplementary Figure 4.** Suppressed Jagged1-Notch1 signaling with hampered angiogenesis after acute ischemic stroke in T2DM. (A-C) The expression of Ki67 and CD31 was assessed by immunofluorescence staining in ischemic penumbra (Magnification * 200. Scale bar = 100um) and the positive cells of Ki67 and CD31 were calculated (n = 3 per group). (D-E) The gene expression of Jagged1, Notch1, Hes1 and Hes5 was showed in NDI and DI group. (F-G) The expression of Jagged1, Notch1, NICD and Hes1 protein were calculated in NDI group and DI group. * p < 0.05 vs. sham group, ** p < 0.01 vs. sham group, *** p < 0.001 vs. sham group, **** p <0.0001 vs. sham group


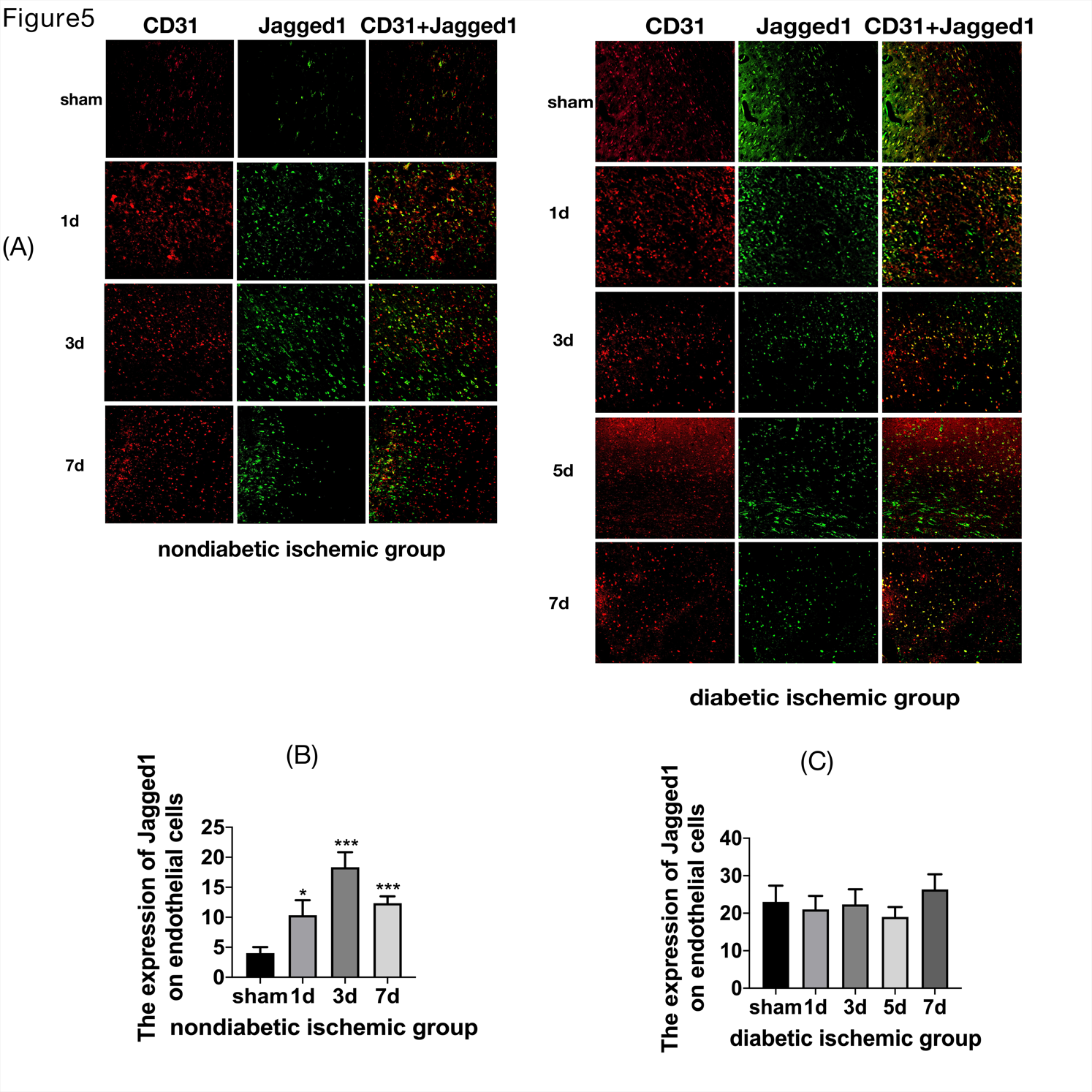


**Supplementary Figure 5.** Suppressed Jagged1-Notch1 signaling after acute ischemic stroke in T2DM. (A-C) The expression of Jagged1 and CD31 was assessed by immunofluorescence staining in ischemic penumbra (Magnification * 200. Scale bar = 100um) and the positive cells of Jagged1 and CD31 were calculated (n = 3 per group). * p < 0.05 vs. sham group, *** p < 0.001 vs. sham group.


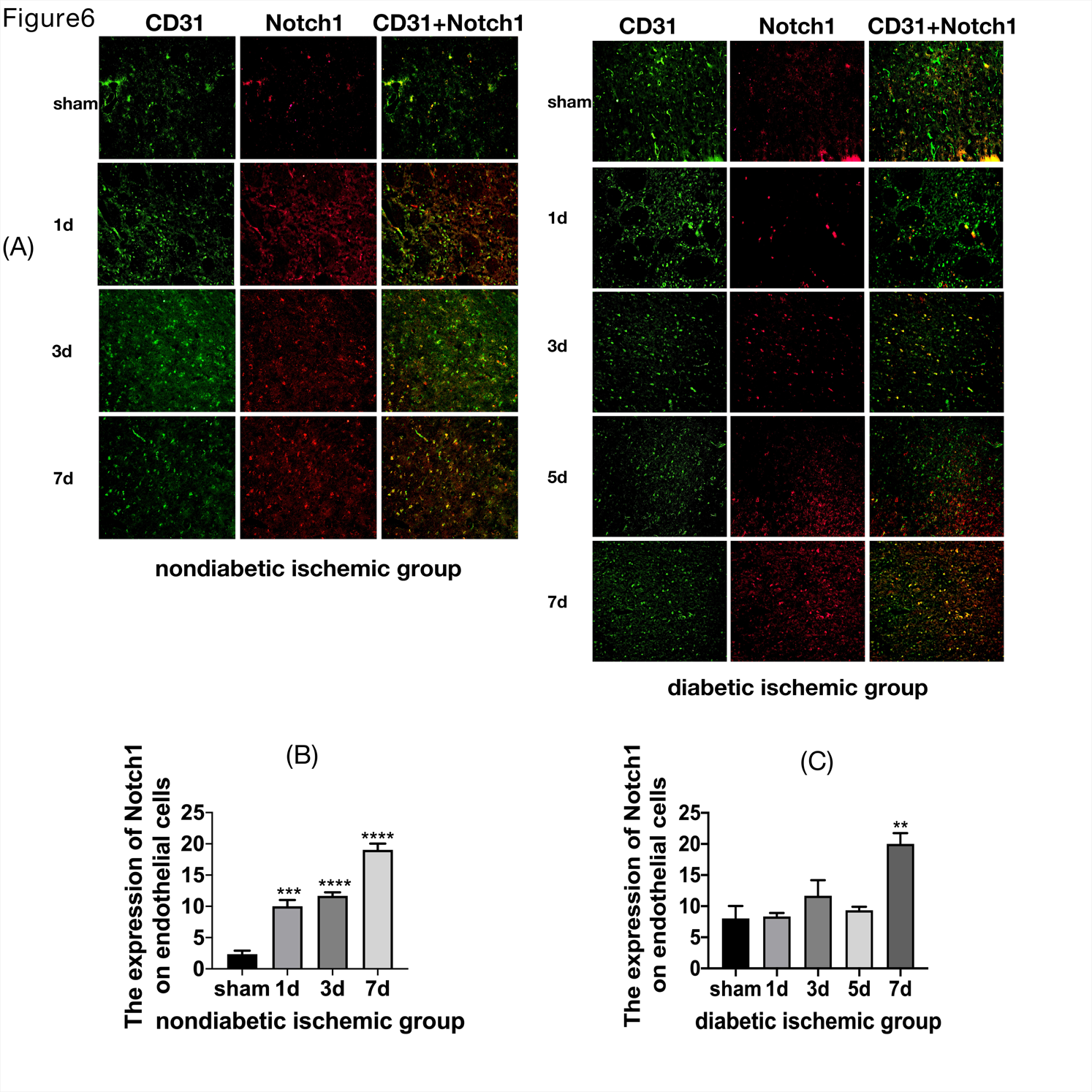


**Supplementary Figure 6.** Suppressed Jagged1-Notch1 signaling after acute ischemic stroke in T2DM. (A-C) The expression of Notch1 and CD31 was assessed by immunofluorescence staining in ischemic penumbra (Magnification * 200. Scale bar = 100um) and the positive cells of Notch1 and CD31 were calculated (n = 3 per group). ** p < 0.01 vs. sham group, *** p < 0.001 vs. sham group, **** p <0.0001 vs. sham group.


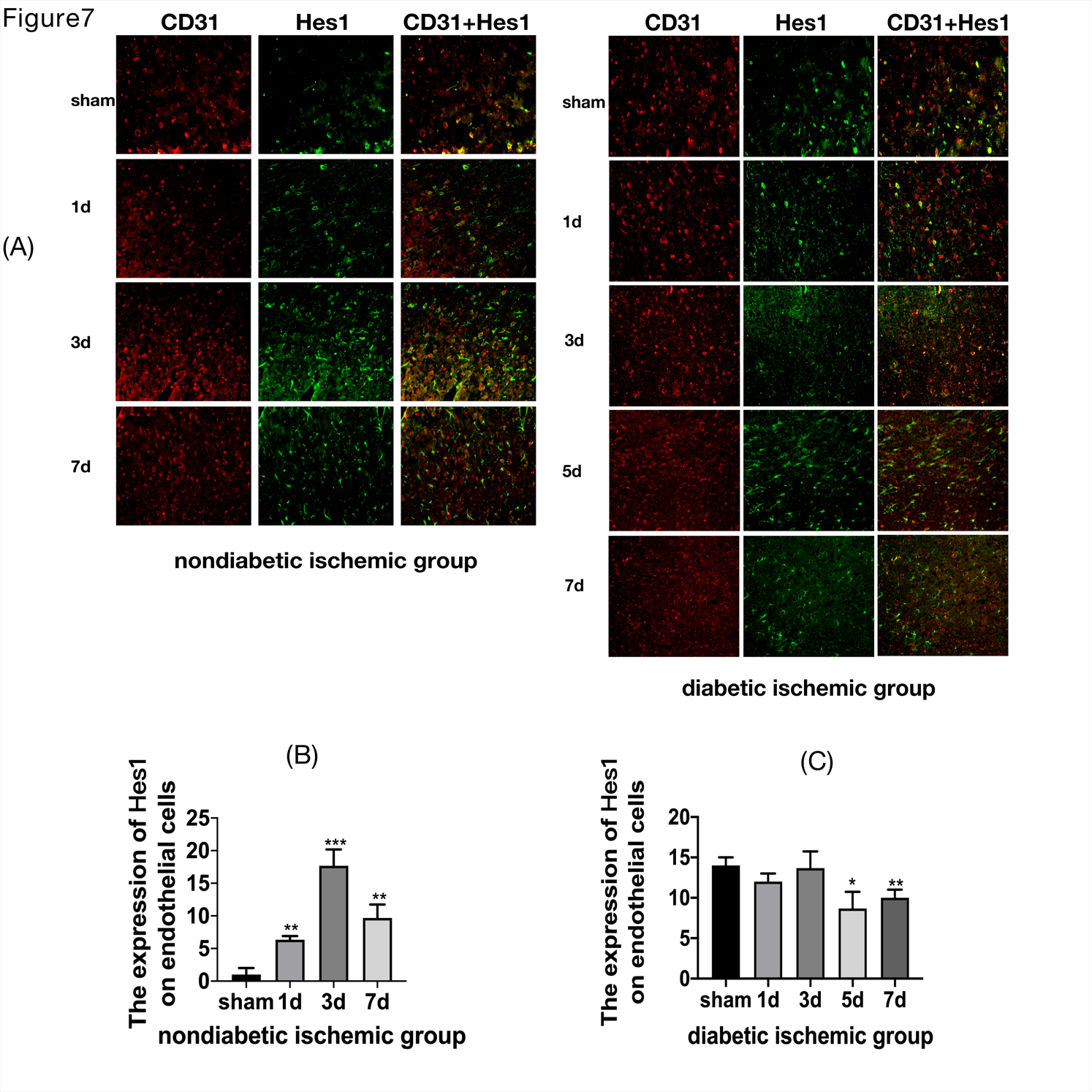


**Supplementary Figure 7.** Suppressed Jagged1-Notch1 signaling after acute ischemic stroke in T2DM. (A-C) The expression of Hes1 and CD31 was assessed by immunofluorescence staining in ischemic penumbra (Magnification * 200. Scale bar = 100um) and the positive cells of Hes1 and CD31 were calculated (n = 3 per group). * p < 0.05 vs. sham group, ** p < 0.01 vs. sham group, *** p < 0.001 vs. sham group.


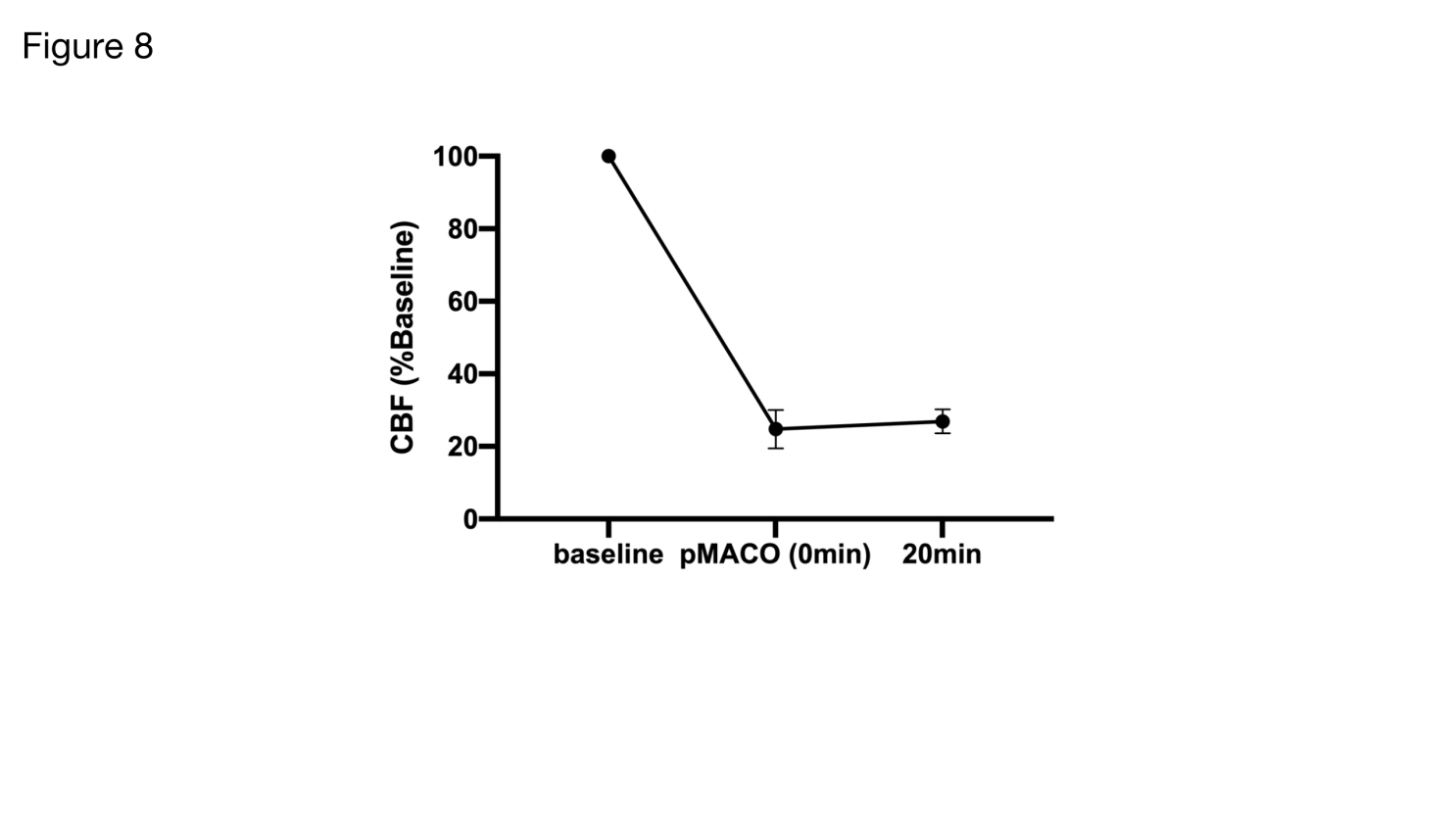


**Supplementary Figure 8.** Using Laser Doppler flowmetry (Perimed, Sweden) monitoring cerebral blood flow (CBF) before, during and 20 minutes after middle cerebral artery occlusion (MCAO).
